# Mast cells are permissive for rhinovirus replication: potential implications for asthma exacerbations

**DOI:** 10.1111/cea.12879

**Published:** 2017-01-26

**Authors:** C. Akoto, D. E. Davies, E. J. Swindle

**Affiliations:** ^1^ Clinical and Experimental SciencesFaculty of MedicineUniversity of SouthamptonUniversity Hospital SouthamptonSouthamptonUK; ^2^ NIHR Southampton Respiratory Biomedical Research UnitUniversity Hospital SouthamptonSouthamptonUK

**Keywords:** asthma, interferon, innate immunity, mast cells, virus

## Abstract

**Background:**

Human rhinoviruses (HRVs) are a major trigger of asthma exacerbations, with the bronchial epithelium being the major site of HRV infection and replication. Mast cells (MCs) play a key role in asthma where their numbers are increased in the bronchial epithelium with increasing disease severity.

**Objective:**

In view of the emerging role of MCs in innate immunity and increased localization to the asthmatic bronchial epithelium, we investigated whether HRV infection of MCs generated innate immune responses which were protective against infection.

**Methods:**

The LAD2 MC line or primary human cord blood‐derived MCs (CBMCs) were infected with HRV or UV‐irradiated HRV at increasing multiplicities of infection (MOI) without or with IFN‐β or IFN‐λ. After 24 h, innate immune responses were assessed by RT‐qPCR and IFN protein release by ELISA. Viral replication was determined by RT‐qPCR and virion release by TCID_50_ assay.

**Results:**

HRV infection of LAD2 MCs induced expression of IFN‐β, IFN‐λ and IFN‐stimulated genes. However, LAD2 MCs were permissive for HRV replication and release of infectious HRV particles. Similar findings were observed with CBMCs. Neutralization of the type I IFN receptor had minimal effects on viral shedding, suggesting that endogenous type I IFN signalling offered limited protection against HRV. However, augmentation of these responses by exogenous IFN‐β, but not IFN‐λ, protected MCs against HRV infection.

**Conclusion and Clinical Relevance:**

MCs are permissive for the replication and release of HRV, which is prevented by exogenous IFN‐β treatment. Taken together, these findings suggest a novel mechanism whereby MCs may contribute to HRV‐induced asthma exacerbations.

## Introduction

Asthma is a complex and heterogeneous chronic respiratory disease affecting over 300 million people worldwide 1. It is characterized by airway inflammation and variable and reversible airway obstruction resulting in symptoms of wheeze, chest tightness and shortness of breath 2. Human rhinoviruses (HRVs) are a major risk factor for asthma development in early life 3 and are the major cause of viral‐induced exacerbations of asthma 4, 5. There are over 100 serotypes of HRV which fall into three species (HRV‐A, HRV‐B and HRV‐C) that use different receptors to enter their target cell 6. The majority of HRV‐A and all of HRV‐B use ICAM‐1 (major group), the remaining HRV‐A use members of the low‐density lipoprotein receptor (LDLR) family (minor group) 7, and the recently discovered HRV‐C species uses cadherin‐related family member 3 (CDHR3) 8. A number of cell types are susceptible to HRV infection, including the airway epithelium, which is the principal site of HRV replication 9, and innate immune cells such as macrophages 10, 11 and dendritic cells 12. These cells detect HRVs via a number of pattern recognition receptors (PRRs), which trigger immune responses including the expression of type I and III IFNs, cytokines and chemokines 13, 14. IFNs induce a range of IFN‐stimulated genes (ISGs) via which they mediate their antiviral activities 15. Deficiencies in IFN production have been reported in bronchial epithelial cells and bronchoalveolar lavage macrophages from asthmatic subjects 16, 17, 18; however, this may relate to severity of disease 19, 20.

Mast cells (MCs) are tissue‐resident innate immune cells found predominantly in vascularized tissues, which interface with the external environment including the skin, gastrointestinal tract and the airways 21. They are bone marrow‐derived haematopoietic cells, which are classically associated with the early‐phase allergic reaction in asthma 22. In asthma, MC numbers are increased in the bronchial epithelium, submucosal glands and bronchial smooth muscle where they have an activated phenotype 23, 24, 25. Recent studies have shown that MC location and phenotype change with increasing asthma severity and are closely related to Th2 biomarkers 26, 27, 28, 29. During experimental HRV infection, MC numbers are increased in the bronchial epithelium of asthmatics 30 putting them in close proximity to the major site of HRV replication where they may contribute to viral immunity during HRV‐induced asthma exacerbations.

Aside from their well‐established roles in allergic disorders, MCs are ideally placed within the airways to act as sentinels of the immune system and protect the body from invading pathogens. MCs express a range of PRRs including TLRs, retinoic acid‐inducible gene (RIG)‐I‐like family receptors and NOD‐like receptors and have roles in immunity to parasite and bacterial infections 31. Following bacterial exposure, MCs release cytokines and chemokines that recruit and activate effector cells including neutrophils which clear the infection and dendritic cells which induce acquired immune responses 32. While MCs play a key role in innate immunity towards parasites and bacteria, their role in viral immunity is less clear. In response to dengue virus, MCs release chemokines and cytokines, which recruit NK cells and cytotoxic T cells 33, 34, 35, and MCs release IFNs following TLR3 activation, influenza virus, respiratory syncytial virus (RSV) 36 and sendai virus infection (a murine virus used to model human parainfluenza virus infection) 37. Following RSV infection, cord blood‐derived MCs (CBMCs) increase type I IFN expression and release CXCL10 [interferon gamma‐induced protein 10 (IP‐10)], CCL5 [regulated on activation, normal T cell expressed and secreted (RANTES)] and CCL4 [macrophage inflammatory protein‐1β (MIP‐1β)], which are associated with NK cell, T cell and monocyte recruitment, respectively 38. However in mouse models of influenza A virus (IAV) infection, MC‐deficient mice are less susceptible to influenza‐induced weight loss than MC knock‐in mice, suggesting MC activity was detrimental during the infection 39. Regarding HRV, there is only a single study, which has demonstrated the immature HMC‐1 cell line can be infected with HRV14; however, this did not trigger any responses unless the cells were also challenged with other stimuli 40.

In view of the importance of HRV‐induced asthma exacerbations and the localization of MCs within asthmatic airways 25, we have investigated the innate immune response of mature LAD2 MCs or primary CBMCs to major and minor group HRV exposure and determined their susceptibility to infection. Exposure of human MCs to HRV induced increases in type I IFNs, ISGs and inflammatory mediators. However, MCs were susceptible to HRV infection and were permissive for viral replication and production of infectious virus particles. Exogenous IFN‐β treatment was protective against infection and this may have important consequences in moderate/severe asthma where epithelial IFN responses are impaired 16, 41.

## Methods

### Reagents

Human IFN‐β and IFN‐λ1 were purchased from the National Institute for Biological Standards and Control (NIBSC, Potters Bar, UK), and stem cell factor (SCF), IL‐6 and IL‐3 were purchased from Peprotech (London, UK). Mouse anti‐human IFN‐α/βR chain 2 antibody (anti‐IFNAR2, IgG2a, clone MMHAR2) was purchased from PBL assay science (Piscataway, USA) and mouse IgG2a isotype control was purchased from R&D systems (Abingdon, UK). Unless otherwise stated, all other cell culture medium and reagents were purchased from Thermo Fisher Scientific (Inchinnan, UK).

### Cell culture

The human MC line LAD2 42 was maintained in StemPro^®^‐34 serum‐free medium supplemented with SCF (100 ng/mL), l‐glutamine (2 mM), penicillin (100 U/mL) and streptomycin (100 μg/mL). Culture medium was replenished weekly by hemidepletion. Cells were > 99% positive for CD117 and FcεRI expression as determined by flow cytometry.

Human cord blood‐derived MCs (CBMCs) were derived from CD34^+^ cord blood mononuclear cells (Stemcell Technologies, Grenoble, France). CD34^+^ cells were maintained in StemPro^®^‐34 medium supplemented with IL‐3 (30 ng/mL, 1st week only), IL‐6 (100 ng/mL) and SCF (100 ng/mL) for a minimum of 8 weeks. CBMCs were > 99% pure by flow cytometric analysis of CD117 expression.

The human bronchial epithelial cell (BEC) line, 16HBE‐14o‐ (16HBE) 43, was maintained in MEM‐GlutaMax™ supplemented with FBS (10% v/v), penicillin (100 U/mL) and streptomycin (100 μg/mL) (16HBE medium) and seeded in six‐well plates precoated with collagen (30 μg/mL; Advanced BioMetrix, San Diego, USA) prior to use in experiments.

### Human rhinovirus stocks

RV16 (major group) and RV1B (minor group) stocks were generated using HeLa cells as previously described 44. Virus titres of cell‐free supernatant stocks were determined by tissue culture infective dose 50% (TCID_50_)/mL according to the Spearman–Karber method. Controls of UV‐irradiated HRV (1200 mJ/cm^2^ on ice for 50 min) were included in all experiments.

### Human rhinovirus infection and treatment of cells

LAD2 MCs or CBMCs (0.5 × 10^6^ cells/mL) were infected with RV16 or RV1B (multiplicity of infection (MOI) 0.3, 3 or 7.5) or UV‐HRV (MOI 7.5) or treated with HRV infection medium [MEM‐Glutamax™plus FBS (4% (v/v)), nonessential amino acids (1% (v/v)), penicillin (100 U/mL)/streptomycin (100 μg/mL), HEPES (16 mm), NaHCO_3_ (0.12% (v/v)), tryptose (0.118% (v/v)) and MgCl_2_ (0.3 mM)] (IM; mock infection) as a control for 1 h before washing with StemPro^®^‐34 medium to remove excess virus. Cells were then incubated at 37°C and cell‐free supernatants and cell pellets harvested at 24 h. Cell viability was determined by trypan blue exclusion. As a positive control, 16HBE cells were infected with RV16 (MOI 0.3, 3 or 7.5), UV‐RV16 (MOI 7.5) or HRV infection medium following overnight starvation in 16HBE medium with 2% (v/v) FBS. In selected experiments, cells were treated with IFN‐β (100 IU/mL) or IFN‐λ1 (100 IU/mL) at the time of RV16 infection or pretreated with anti‐IFNAR2 antibody (1 μg/mL) or IgG2a isotype control (1 μg/mL) 1 h prior to RV16 infection.

### Real‐time quantitative PCR

Total RNA was isolated [Trizol reagent or Qiagen RNeasy mini kit (Manchester, UK)] and treated for genomic DNA contamination prior to quantification and reverse transcription to cDNA (Primerdesign, Southampton, UK). For each quantitative RT‐PCR (RT‐qPCR), cDNA (12.5 ng) was mixed with PCR master mix containing primer/fluorogenic probes (*IFNB1*,* IFNL1*, interferon regulatory factor‐7 (*IRF7*), MX dynamin‐like GTPase 1 (*MX1*), melanoma differentiation‐associated gene 5 (*MDA5*), *CXCL10*,* CCL5*, RV16, RV1B, cadherin‐related family member 3 (*CDHR3*) or the housekeeping genes (HKGs) *GAPDH* and ubiquitin C (*UBC*) for detection of specific amplification products or primer/SYBR green intercalating dye (2′‐5′‐oligoadenylate synthase 1 (*OAS1*) for detection of double‐stranded amplification products as designed by the manufacturer [Primerdesign, Southampton, UK, or Thermo Fisher Scientific (CDHR only)]. All reactions were performed in duplicate for 50 cycles and gene expression analysed using a real‐time PCR iCycler (Bio‐Rad, Hemel Hempstead, UK). For SYBR green detection‐based reactions, melt curves were performed to ensure single PCR product formation. Gene expression was normalized to the geometric means of HKGs and fold changes calculated relative to UV‐HRV controls according to the ^ΔΔ^Ct method and expressed as 2^‐ΔΔCt^. Viral RNA copy number was determined against a standard curve of known copies of RV16 or RV1B (Primerdesign, Southampton, UK).

### ELISA

IFN‐β and IFN‐λ protein was quantified in concentrated cell‐free supernatants by ELISA according to the manufacturer's instructions (IFN‐λ 1/3; R&D systems, Abingdon, UK. IFN‐β; MSD, Gaithersburg, MD, USA). Supernatants were concentrated (4×) using 3000 nominal MW limit ultrafiltration units (Merck Millipore, Watford, UK).

### Statistical analysis

Paired nonparametric data were analysed with Friedman repeated‐measures one‐way anova by ranks with Dunn's correction for multiple comparisons or Wilcoxon signed rank test for matched pair comparisons. Unpaired nonparametric data were analysed with Kruskal–Wallis one‐way anova with Dunn's correction for multiple comparisons or Mann–Whitney ranked sum test for matched pair comparisons. Data are presented as box and whisker plots showing the median, interquartile range and minimum and maximum values or as floating bars showing median and range. Normalized data were analysed by Student's *t*‐test and are presented as mean ± SEM. All data were analysed using GraphPad Prism (GraphPad Software, Inc., San Diego, CA, USA). *P* ≤ 0.05 was considered statistically significant.

## Results

### The human mast cell line LAD2 mounts an innate immune response to human rhinovirus infection

**Figure 1:**
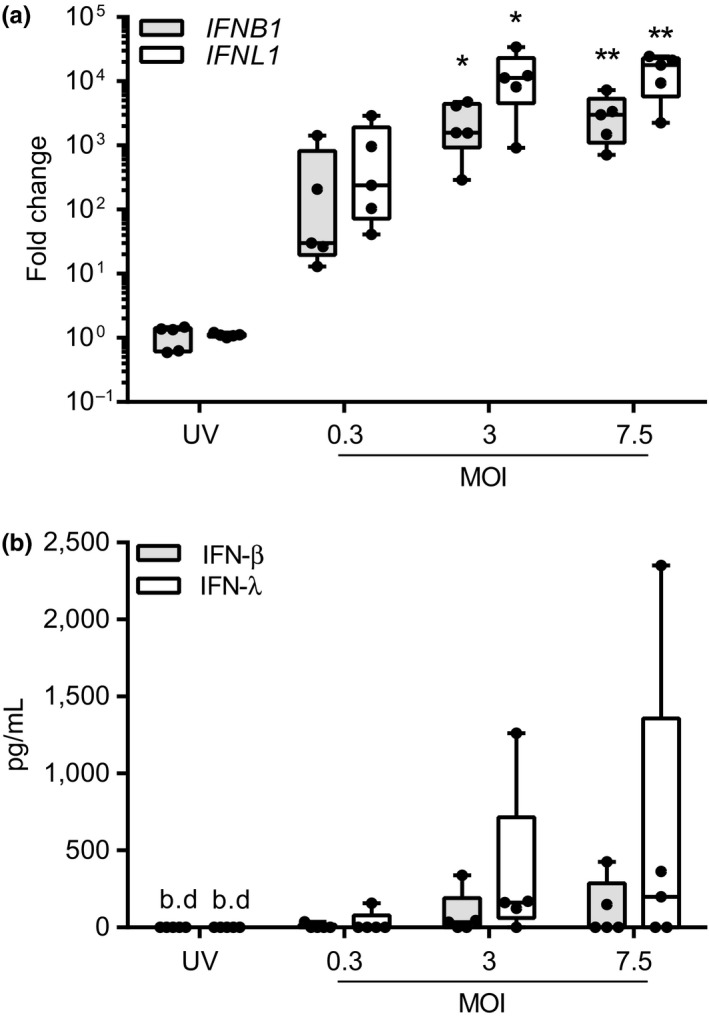
RV16‐induced IFN responses in LAD2 MCs. LAD2 MCs were exposed to RV16 MOI 0.3, 3 or 7.5 or UV‐RV16 MOI 7.5 (control). Cell pellets and cell‐free supernatants were harvested for gene and protein expression by RT‐qPCR and ELISA, respectively. (a) *IFNB1* and *IFNL1* mRNA expression 24 h post‐RV16 infection, *n* = 5. (b) IFN‐β and IFN‐λ protein expression 24 h post‐RV16 infection, *n* = 5. Results are box and whisker plots showing the median, interquartile range and min and max values, *P ≤ 0.05, **P ≤ 0.01 vs. UV‐RV16. MOI, multiplicity of infection. b.d., below limit of detection.

To investigate the role of MCs in HRV immunity, LAD2 MCs were exposed to HRV or UV‐HRV (as a control) and innate immune responses assessed by RT‐qPCR after 24 h. Exposure to the major group virus, RV16, resulted in a significant MOI‐dependent increase in mRNA expression of the type I and type III IFNs, *IFNB1* and *IFNL1*, respectively (Fig. 1a). There was also a trend for increased IFN‐β and IFN‐λ protein release, as detected by ELISA, but this failed to reach statistical significance (Fig. 1b). There was minimal induction of IFN mRNA or protein with UV‐HRV MOI 7.5 [median fold change, *IFNB1*, 1.3 (IQR 0.6–1.4), *IFNL1*, 1.1 (IQR 1.0–1.2)] or mock infection (median fold change, *IFNB1*, 1.2 (IQR 0.9–1.8), *IFNL1*, 2.3 (IQR 0.6–11), data not shown), indicating that virus replication was required to induce the observed responses. In control experiments, RV16 did not induce MC degranulation (Fig. S1). Similar results were obtained with RV1B (minor group virus; Fig. S2a).

**Figure 2:**
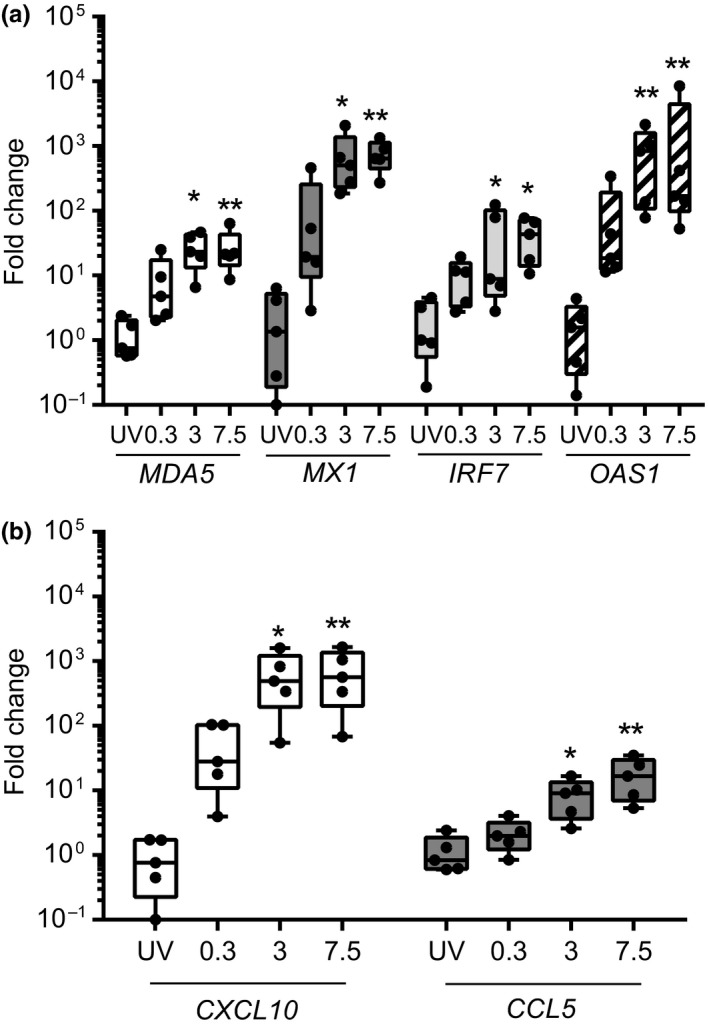
RV16‐induced innate immune responses in LAD2 MCs. LAD2 MCs were exposed to RV16 at MOI 0.3, 3 or 7.5 or UV‐RV16 MOI 7.5 (control). Cell pellets were harvested for gene expression by RT‐qPCR. (a) mRNA expression of interferon‐stimulated genes (*MDA5*,*MX1*,*IRF7* and *OAS1*) and (b) chemokines (*CXCL10* and *CCL5*) 24 h post‐RV16 infection. Results are box and whisker plots showing the median, interquartile range and min and max values, *n* = 5, *P ≤ 0.05, **P ≤ 0.01 vs. UV‐RV16. MOI, multiplicity of infection.

In parallel with the upregulation of IFNs, we also observed significant upregulation of antiviral genes following exposure of LAD2 MCs to HRV. This included the MOI‐dependent induction of *MDA5*,* MX1*,* IRF7* and *OAS1* following exposure to RV16 (Fig. 2a). Additionally mRNA transcripts for the inflammatory mediators *CXCL10* and *CCL5* were also induced (Fig. 2b). In all cases, induction of ISG transcripts was dependent on viral replication as a lack of induction was observed with mock infection or UV‐HRV (MOI 7.5) controls. Similar results were obtained with RV1B (Fig. S2b,c).

### The human mast cell line LAD2 is permissive for human rhinovirus replication and releases infectious virus particles

**Figure 3:**
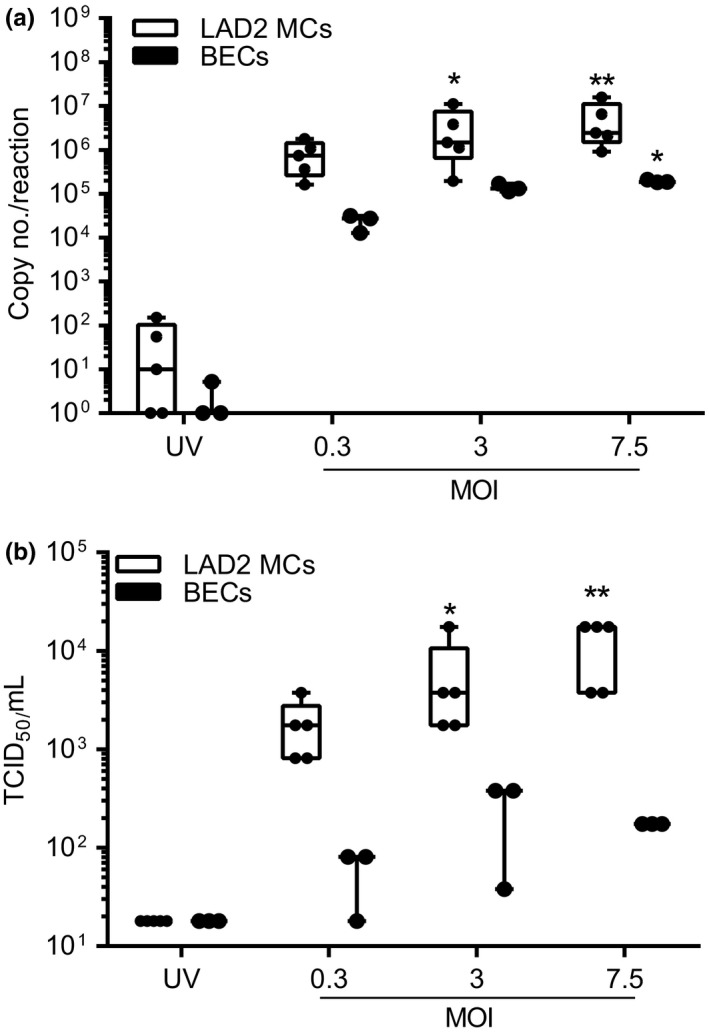
Comparison of the replication and release of infectious RV16 from LAD2 MCs and bronchial epithelial cells (BECs). LAD2 MCs and BECs were exposed to RV16 MOI 0.3, 3 or 7.5 or UV‐RV16 MOI 7.5 (control). Cell pellets and cell‐free supernatants were harvested for viral RNA and infectious virus particles by RT‐qPCR and TCID_50_ assay, respectively. (a) RV16 copy number and (b) TCID_50_/mL 24 h post‐RV16 infection. Results are box and whisker plots showing the median, interquartile range and min and max values, *n* = 5 (LAD2), *n* = 3 (BECs), *P ≤ 0.05, **P ≤ 0.01 vs. UV‐RV16. MOI, multiplicity of infection.

Our data demonstrated that the innate immune responses of LAD2 MCs to HRV was dependent on viral replication as these responses were not observed using the replication deficient UV‐HRV control or mock infection media. Therefore, we used RT‐qPCR to assess viral copy number in MCs and compared this to HRV‐infected BECs, which are the main target for HRV replication. RV16 exposure resulted in a significant MOI‐dependent increase in viral RNA (vRNA) in LAD2 MCs compared with UV‐HRV MOI 7.5 [median, 10 copies (IQR 0–103)] or mock infection (median, four copies (IQR 0–65); UV‐HRV vs. MOI 3, *P* = 0.02, UV‐HRV vs. MOI 7.5, *P* = 0.002) (Fig. 3a). Copies of RV16 RNA in LAD2 MCs exceeded those seen using BECs infected with RV16 in the same experiment. This permissiveness for viral replication prompted us to investigate whether LAD2 MCs, like BECs, had the potential to release infectious virus particles. TCID_50_ assay revealed a significant MOI‐dependent increase in the release of infectious RV16 virions from LAD2 MCs [18 TCID_50_/mL for UV‐HRV MOI 7.5 compared with 3768 TCID_50_/mL for HRV MOI 3 (*P* = 0.03) and 17 461 TCID_50_/mL for HRV MOI 7.5, (*P* = 0.002)] (Fig. 3b). Similar results were obtained with RV1B (Fig. S2d). Of note, there was no significant difference in cell viability for RV16‐ or RV1B‐infected LAD2 MCs compared with mock‐infected control cells (Fig. S3a,b).

### Primary human mast cells mount an innate immune response to human rhinovirus infection and also release infectious virus particles

**Figure 4:**
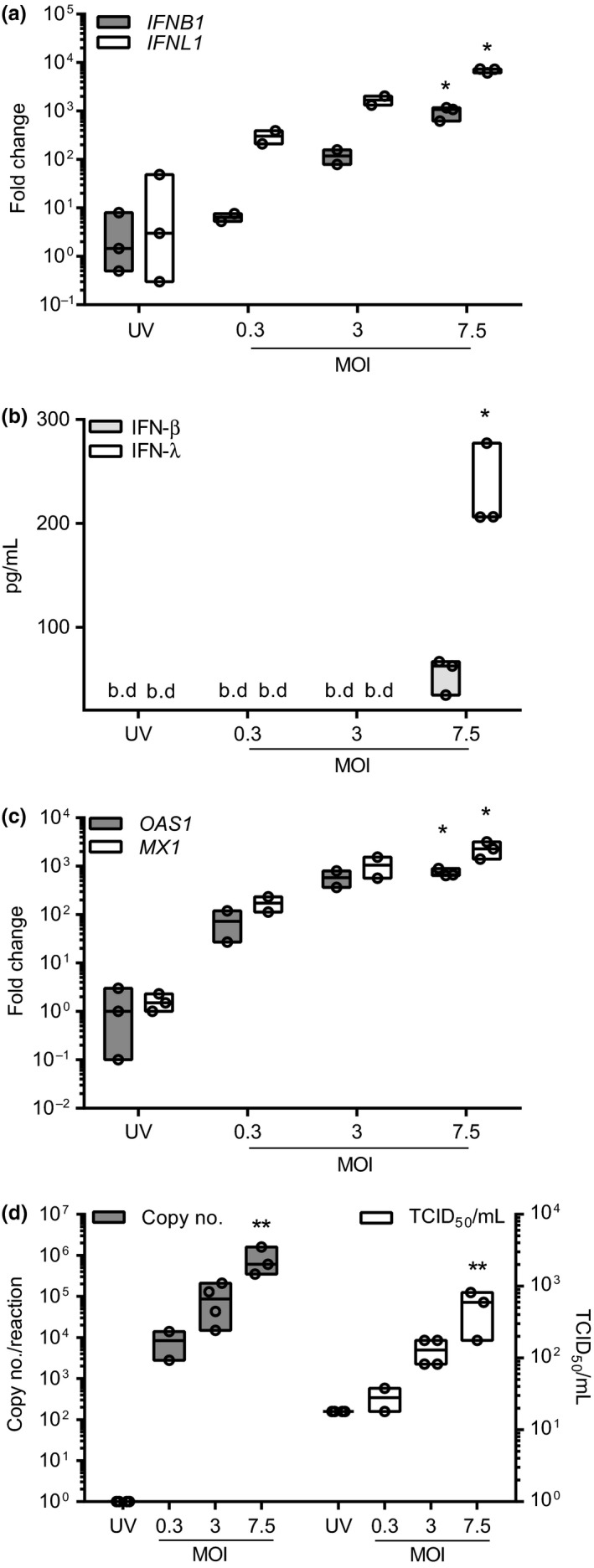
Innate immune responses and release of infectious RV16 from CBMCs. CBMCs were exposed to RV16 at MOI 0.3, 3 or 7.5 or UV‐RV16 MOI 7.5 (control). Twenty‐four h post‐infection cell pellets were harvested for gene expression and viral RNA by RT‐qPCR and cell‐free supernatants for protein expression by ELISA and infectious virus particles by TCID_50_ assay. (a) *IFNB1* and *IFNL1* mRNA expression. (b) IFN‐β and IFN‐λ protein expression. (c) *OAS1* and *MX1* mRNA expression. (d) RV16 copy number and TCID_50_/mL. Results are floating bars representing the median with min and max values, *n* = 2–4, *P ≤ 0.05, **P ≤ 0.01, vs. UV‐RV16 (*n* = 3–4). MOI, multiplicity of infection. b.d., below limit of detection.

Having established the responses of LAD2 MCs to HRV infection, we next investigated the response of primary human CBMCs to RV16 exposure. CBMCs exposed to RV16 for 24 h upregulated the expression of *IFNB1* and *IFNL1* mRNA transcripts (Fig 4a), which was confirmed at the protein level for IFN‐λ but not IFN‐β (Fig. 4b). There was also a significant upregulation of the ISGs *MX1* and *OAS1* in RV16‐infected CBMCs (Fig. 4c). The upregulation of IFNs and ISGs required replication competent virus as UV‐irradiated HRV failed to induce mRNA transcripts (Fig. 4). Crucially, CBMCs were also susceptible for the replication and release of infectious RV16 as observed by a significant increase in vRNA transcripts and virion release (Fig. 4d). As with LAD2 MCs, cell viability was unaffected by RV16 infection (Fig. S3c).

### Primary human mast cells are protected from RV16 infection by exogenous IFN‐β

**Figure 5:**
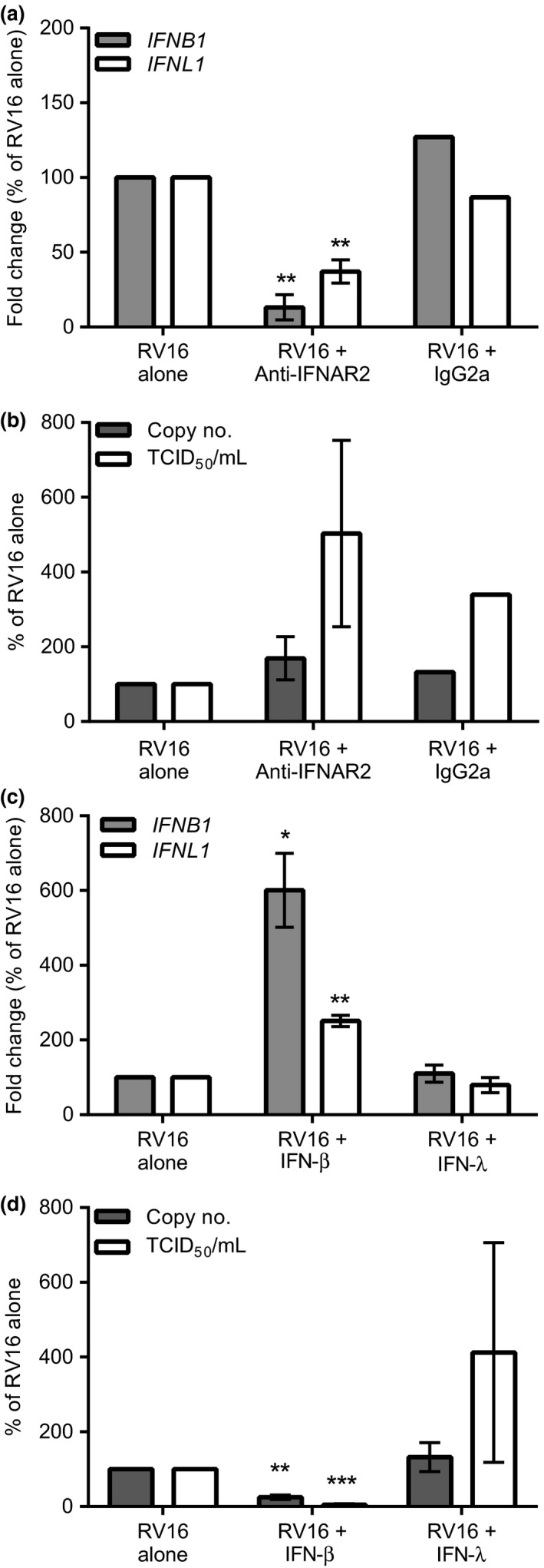
Type I IFN receptor blockade and the effect of exogenous IFN treatment of CBMCs during RV16 infection. CBMCs were pretreated with anti‐IFNAR2 (1 μg/mL) or IgG2a isotype (1 μg/mL) prior to RV16 MOI 7.5 or UV‐RV16 MOI 7.5 exposure. Twenty‐four h post‐infection cell pellets were harvested for gene expression and viral RNA by RT‐qPCR and cell‐free supernatants were harvested for infectious virus particles by TCID_50_ assay. (a) *IFNB1* and *IFNL1* mRNA expression. (b) RV16 copy number and TCID_50_/mL. CBMCs were exposed to RV16 MOI 7.5 or UV‐RV16 MOI 7.5 in the presence or absence of IFN‐β (100 IU/mL) or IFN‐λ (100 IU/mL). (c) *IFNB1* and *IFNL1* mRNA expression. (d) RV16 copy number and TCID_50_/mL. Results are % of control (RV16 alone) means ± SEM,* n* = 2–3. *P ≤ 0.05, **P ≤ 0.01 vs. control (*n* = 3). Anti‐IFNAR2, anti‐IFN‐α/βR 2 antibody; MOI, multiplicity of infection.

Despite the induction of IFNs and antiviral responses, HRV infection of CBMCs still resulted in release of infectious virus particles. We hypothesized that this was due to low levels of endogenous IFN‐β protein induced during HRV infection providing inadequate protection. Therefore, to test the extent of protection by endogenous type I IFNs, we pretreated CBMCs with a type I IFN receptor‐blocking antibody (anti‐IFNAR2) prior to HRV infection. Although this resulted in a significant reduction in HRV‐dependent expression of IFN (*IFNB1* and *IFNL1*) and ISG (*OAS1* and *MX1*, data not shown) mRNAs compared with control (Fig 5a), it had minimal effects on vRNA levels and there was only a trend for increased virion release (Fig. 5b). This implied a minimal protective effect of endogenous type I IFN signalling against HRV replication.

We therefore examined whether we could augment antiviral immune responses of CBMCs by the addition of IFNs to the cultures. CBMCs responded to exogenous IFN‐β with significantly increased expression of *IFNB1* and *IFNL1* mRNA above that observed with HRV alone (Fig. 5c). In contrast, IFN‐λ was without effect suggesting IFN‐β is a key driver of type I and type III IFN responses in these cells; this was confirmed by showing up‐regulation of IFN‐λ protein by IFN‐β (Fig. S4a). Most importantly, exogenous IFN‐β, but not IFN‐λ, protected CBMCs from viral replication and release of infectious virus particles (Fig. 5d). The induction of IFNs and suppression of viral replication mediated by IFN‐β was prevented in the presence of anti‐IFNAR2 antibody (Fig. S4b,c) confirming the blocking antibody effectively suppressed type I IFN signalling.

## Discussion

Viral infections caused by HRVs are a major cause of asthma pathogenesis and exacerbation 4, 5. MCs localize to the bronchial epithelium in asthma according to disease severity 28 and are recruited to the bronchial epithelium following HRV infection 30. While MCs are classically associated with early‐phase allergic reactions in asthma, their role in viral immunity is unclear. Here, we demonstrate that human MCs exposed to either a major or minor group HRV mount innate immune responses including the induction of type I and III IFNs and ISGs. Despite this, MCs were permissive for viral replication and production of infectious virus particles, suggesting that the endogenous immune response was insufficient to limit HRV replication. Consistent with this, exogenous IFN‐β, but not IFN‐λ, was sufficient to prevent the release of infective virus particles and protect MCs against HRV infection. The failure of IFN‐λ to exert an antiviral immune response suggests that MCs like other hematopoietic cells lack receptors for type III interferons 37 whose expression is mainly restricted to cells of epithelial origin 45.

Despite mounting innate immune responses to HRV infection, MCs were permissive for HRV replication and released infectious viral particles which increased with increasing MOI in both the human MC line and primary CBMCs and confirms a previous study using the immature HMC‐1 cell line and HRV14 40. However, this study was limited to investigating a single RV14 infection titre (10^4^ TCID_50_ U/mL) of unknown MOI and focused on the modulation of PMA/ionomycin‐ or IgE/anti‐IgE‐dependent histamine and cytokine release 40. Our study has expanded the findings of Hosoda *et al*. by demonstrating an MOI‐dependent effect on virion release and induction of antiviral and pro‐inflammatory responses of the mature LAD2 cell line and primary MCs following infection with both a major and minor group HRV. The release of infectious HRV virions by MCs is in contrast to infection of MCs by other respiratory viruses including reovirus, RSV and IAV which are capable of infecting human MCs and inducing innate immune responses; however, there is little or no release of virus progeny 33, 38, 46. For instance, plaque assays of RSV infected CBMC supernatants confirm a lack of productive RSV infection of CBMCs 38. It has been shown previously that CBMCs can support replication of dengue virus with release of infectious virus particles; however, this process is antibody dependent 47. HRV replication and release of infectious virus particles from epithelial cells typically result from lysis 14, but here we show that virus shedding from MCs is not associated with significant cell death. Nonlytic virus shedding has been reported for other picornaviruses including poliovirus 48, 49, 50 and may be a mechanism by which infective HRV particles are released from MCs.

As MCs accumulate in the bronchial epithelium in asthma, they have the potential to come into close proximity with HRV during infection of the bronchial epithelium. Our findings that MCs are permissive for HRV replication and that virus shedding was not associated with significant cell death suggest that MCs may act as reservoirs for HRV and this may potentiate HRV‐induced asthma exacerbations. This mechanism seems unique among common respiratory viruses and may help to explain the high association of HRV infection and asthma exacerbations, with MCs playing a novel pathological role. This may be particularly relevant in HRV‐induced asthma exacerbations of difficult‐to‐treat severe asthma patients where MC numbers 28 and MC‐specific mediators 29 are both increased. During experimental HRV infection of adults, MCs have been demonstrated to accumulate in the bronchial mucosa 30; however, it is not known whether MCs *in vivo* are susceptible to HRV infection. This question may be addressed by performing *in situ* hybridization on bronchial biopsies taken from subjects following experimental HRV infection to determine whether HRV particles localize within MCs. Alternatively, MCs may be isolated from these biopsies via laser capture for the detection of vRNA. In children, HRV infection is implicated in the inception of childhood asthma where infection is a major cause of persistent wheeze in infants and is a major risk factor for asthma development in early life 3. Furthermore, the number of mucosal MCs and reticular basement membrane thickness at age 1 year predicts respiratory morbidity and the use of inhaled corticosteroids at age 3 years 51. This suggests an important interaction between viral infection and MCs leading to allergic inflammation and the development of asthma in young children. While we demonstrated human MCs are permissive for the replication of major and minor group HRVs, it is unlikely that they would be permissive for HRV‐C infection as they do not express CDHR3 (Fig. S5), the cellular receptor for HRV‐C 8.

Following HRV infection, MCs upregulated type I and type III IFNs (IFN‐β and IFN‐λ), PRRs (*MDA5*), ISGs (*IRF7*,* OAS1*,* MX1*) and chemokines (*CXCL10* and *CCL5*). The induction of type I IFNs is likely to be a general response of MCs to virus exposure as these IFNs are also up‐regulated with dengue virus 35, IAV 36, RSV 36 and sendai virus 37. While type III IFN is generated following viral infection by many different cell types 17, 52, 53, reports of virus‐dependent type III IFN expression and release by MCs are limited 37. Although not able to respond to IFN‐λ, the release of these antiviral proteins by MCs may help promote antiviral immunity in epithelial cells, which express IFN‐λ receptors 54. Despite the small amounts of IFN‐β production, the suppression of *IFNB1* and *IFNL1*, as well as the ISGs *OAS1* and *MX1*, following anti‐IFNAR2 treatment of CBMCs, suggests an IFN driven ISG response. ISGs can also be induced via TLR activation 55 and viral dsRNA (including replication intermediates of ssRNA viruses) 56 has been shown to activate MCs via TLR3 36. Therefore, HRV‐induced IFN as well as TLR activation may contribute to the observed ISG induction. Induction of viral sensors and antiviral genes such as *MDA5*,* MX1*,* OAS1* has also been observed in human MCs infected with dengue virus 35 and vaccinia virus 54, but reports of up‐regulation of these genes following infection with respiratory viruses are limited to sendai virus infection, a murine virus used to model human parainfluenza virus infection 37. Exposure of MCs to many different viruses including dengue virus, reovirus and RSV induces the release of cytokines and chemokines, which are speculated to recruit inflammatory cells to help clear the infection 33, 38, 57. In response to reovirus, MCs release CXCL8 which recruits NK cells 33 and CCL3‐5 which recruit a subsets of T cells 34 *in vitro*. Dengue virus infection of mice also results in MC‐dependent recruitment of NK and NKT cells, although the specific mediators involved were not investigated 58. Therefore, effector cells including T cells, NK cells, and DCs may be recruited via MC‐derived chemokines including CXCL10 and CCL5 during HRV infection; however, whether inflammatory cell recruitment would result in viral clearance or contribute to asthma pathology requires further investigation.

Human MCs were highly permissive for HRV infection which appeared to be due to only low‐level production of IFN‐β. Although we did not measure other type I IFNs, we used a type I IFN receptor‐blocking antibody to investigate whether endogenous IFN‐β or IFN‐α made a substantial contribution to defence against HRV. This showed minimal effects on viral replication and a trend for enhanced shedding of infectious HRV particles confirming limited protection by endogenous type I interferons. Therefore, we investigated whether exogenous IFN‐β could boost IFN responses and found significant up‐regulation of *IFNB1*,* IFNL1* and an associated suppression of viral replication and release. IFN also up‐regulates the expression of RIG‐I, MDA5 and TLR3 in MCs suggesting increased detection of HRV by MCs 37. The demonstration that boosting IFN responses can protect human MCs from HRV infection is of significance, as bronchial epithelial IFN responses following HRV infection are impaired in moderate/severe asthma 16, 17, 19, 20. In addition, the localization of MCs in the bronchial epithelium increases with asthma severity suggesting that in severe asthma, MCs are at an increased risk of HRV infection and viral shedding. Exogenous IFN‐β protects asthmatic primary BECs from HRV infection 16, 59 and inhaled IFN‐β has been shown to be particularly effective at reducing symptoms and improving lung function in difficult‐to‐treat asthmatics during naturally occurring viral respiratory infections 41. It is currently unknown whether MCs from asthmatic patients have a more severe defect in their IFN response to HRV infection. Nonetheless, our findings suggest that, in addition to protecting the bronchial epithelium against HRV infection, an inhaled IFN‐β therapy could protect MCs directly and further boost the innate immune response of the bronchial epithelium by the production of IFN‐λ.

In summary, we have shown for the first time that mature LAD2 MCs and primary CBMCs are permissive for the replication and release of HRV, which implicates them in HRV‐induced asthma exacerbation. Furthermore, exogenous IFN‐β is protective against HRV infection and may be particularly relevant in targeting MCs in severe asthma.

## Conflict of interest

Professor Donna Davies is a cofounder of Synairgen and is paid consultancy fees and also has a patent for the use of inhaled interferon beta therapy for virus‐induced exacerbations of asthma and COPD with royalties paid.

## Supporting information


**Figure S1**. HRV does not induce MC degranulation.
**Figure S2.** LAD2 MC response to RV1B exposure.
**Figure S3**. Cell viability following rhinovirus infection of human mast cells.
**Figure S4**. IFN‐b treatment and RV16 exposure of CBMCs and type I IFN receptor blockade.
**Figure S5**. CDHR3 expression following RV16 infection of LAD2 mast cells.
