# Supplementary material for: Mast cells are permissive for rhinovirus replication: potential implications for asthma exacerbations
Source: Clin Exp Allergy. 2017 Jan 26;47(3):351–60. doi: 10.1111/cea.12879 (PMC5396281; doi:10.1111/cea.12879)
Supplement: Supplementary file 1 — Figure S1. HRV does not induce MC degranulation. Figure S2. LAD2 MC response to RV1B exposure. Figure S3. Cell viability following rhinovirus infection of human mast cells. Figure S4. IFN‐b treatment and RV16 exposure of CBMCs and type I IFN receptor blockade. Figure S5. CDHR3 expression following RV16 infection of LAD2 mast cells. [file CEA-47-351-s001.pdf]

Supplemental information

Mast cells are permissive for rhinovirus replication: potential implications for asthma exacerbations

Charlene Akoto[a], Donna E. Davies[a, b], Emily J. Swindle[a, b].

[a]Clinical and Experimental Sciences, Faculty of Medicine, University of Southampton, University Hospital Southampton, Southampton, UK; [b]NIHR Southampton Respiratory Biomedical Research Unit, University Hospital Southampton, Southampton, UK.

Corresponding author: Emily J. Swindle, Rm LF73, MPT 810, South Academic Block, Level F, Academic Unit of Clinical and Experimental Sciences, Faculty of Medicine, University of Southampton, University Hospital Southampton NHS Foundation Trust, Tremona Road, Southampton, SO16 6YD, United Kingdom. Tel: 02381 208975, email: e.j.swindle@soton.ac.uk.

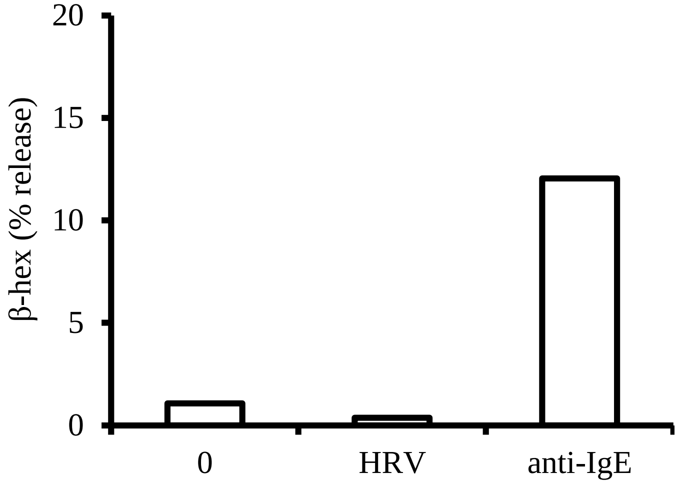

Fig. S1. HRV does not induce MC degranulation.
LAD2 MCs were sensitised with human myeloma IgE (500 ng/mL) overnight prior to stimulation with HRV16 (MOI=1), anti-IgE (10 μg/mL, positive control) or medium (negative control). Cell-free supts were collected after 1h and quantified for net β-hexosaminidase release. Results are a representative experiment performed in duplicate (n=1)

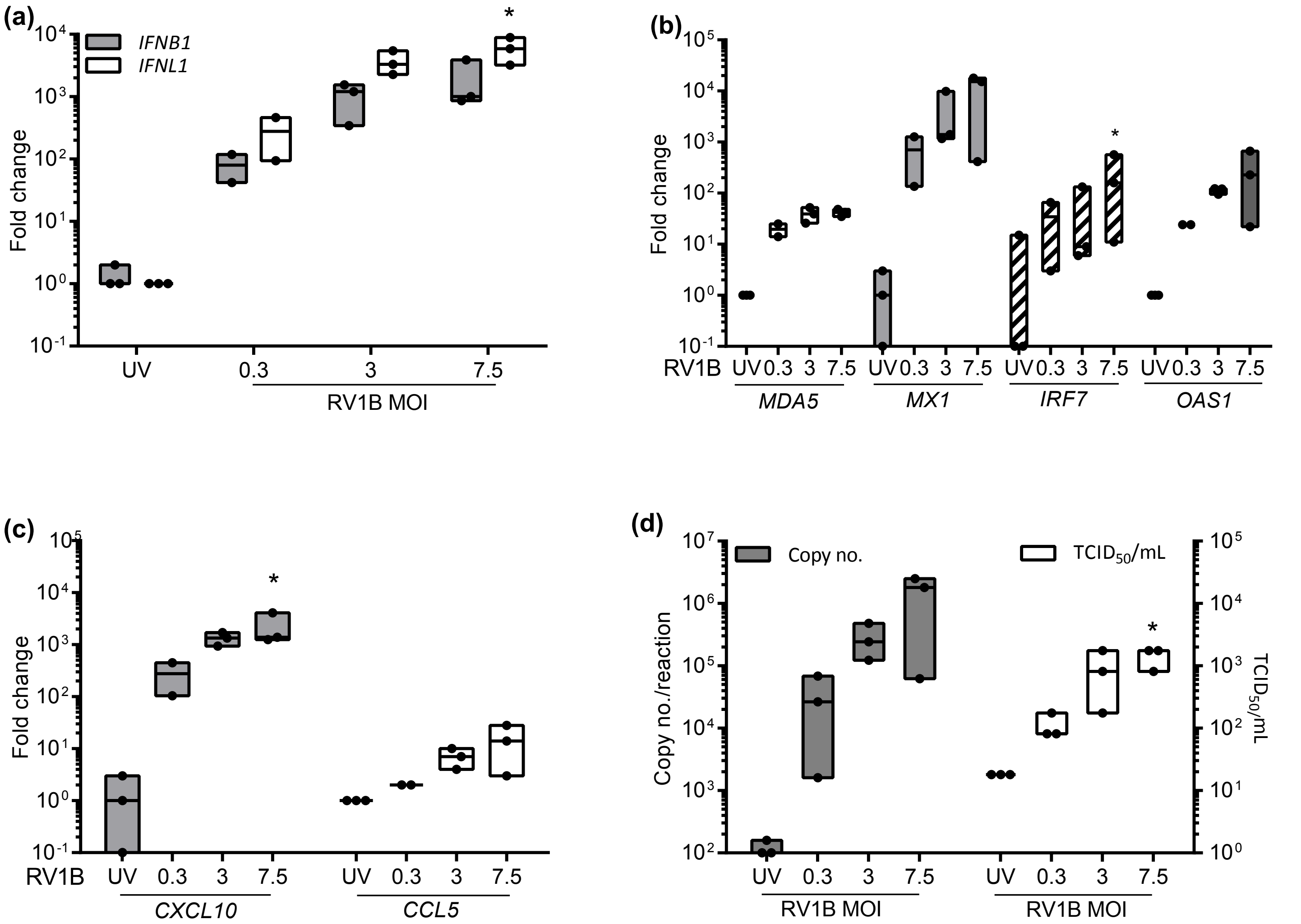

Fig. S2. LAD2 MC response to RV1B exposure. LAD2 MCs were exposed to RV1B at MOI 0.3, 3 or 7.5 or UV-RV1B MOI 7.5 (control). Twenty-four hours post infection cell pellets were harvested for gene expression and viral RNA by RT-qPCR and cell-free supernatants were harvested for infectious virus particles by $TCID_{50}$ assay. (**a**) *IFNB1* and *IFNL1* mRNA expression. (**b**) Interferon stimulated gene mRNA expression (*MDA5*, *MX1*, *IRF7*, *OAS1*). (**c**) Chemokine mRNA expression (*CXCL10*, *CCL5*). (**d**) RV1B copy number and $TCID_{50}$/mL. Floating bars represent the median with min and max values, n=2-3, **P* ≤ 0.05 versus UV-RV1B (n=3). MOI, multiplicity of infection.

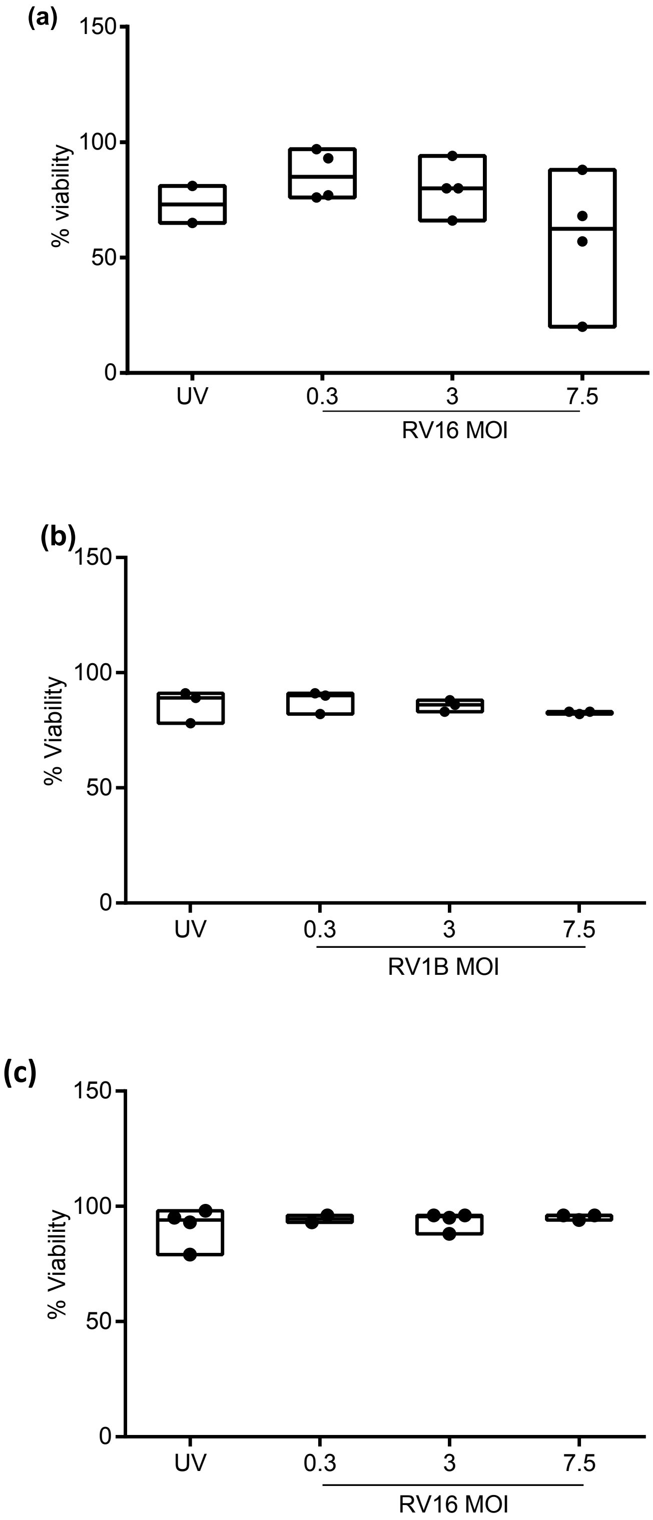

Fig. S3. Cell viability following rhinovirus infection of human mast cells. MCs were exposed to HRV (RV16 or RV1B) at MOI 0.3, 3 or 7.5 or UV-RV16/RV1B MOI 7.5. Twenty-four hours post infection cell viability was determined as a percentage of total cell number by trypan blue exclusion. (**a**) Cell viability of LAD2 MCs following RV16 infection, n=2-5 or (**b**) RV1B infection, n=3. (**c**) Cell viability of CBMCs following RV16 exposure, n=2-4. MOI, multiplicity of infection.

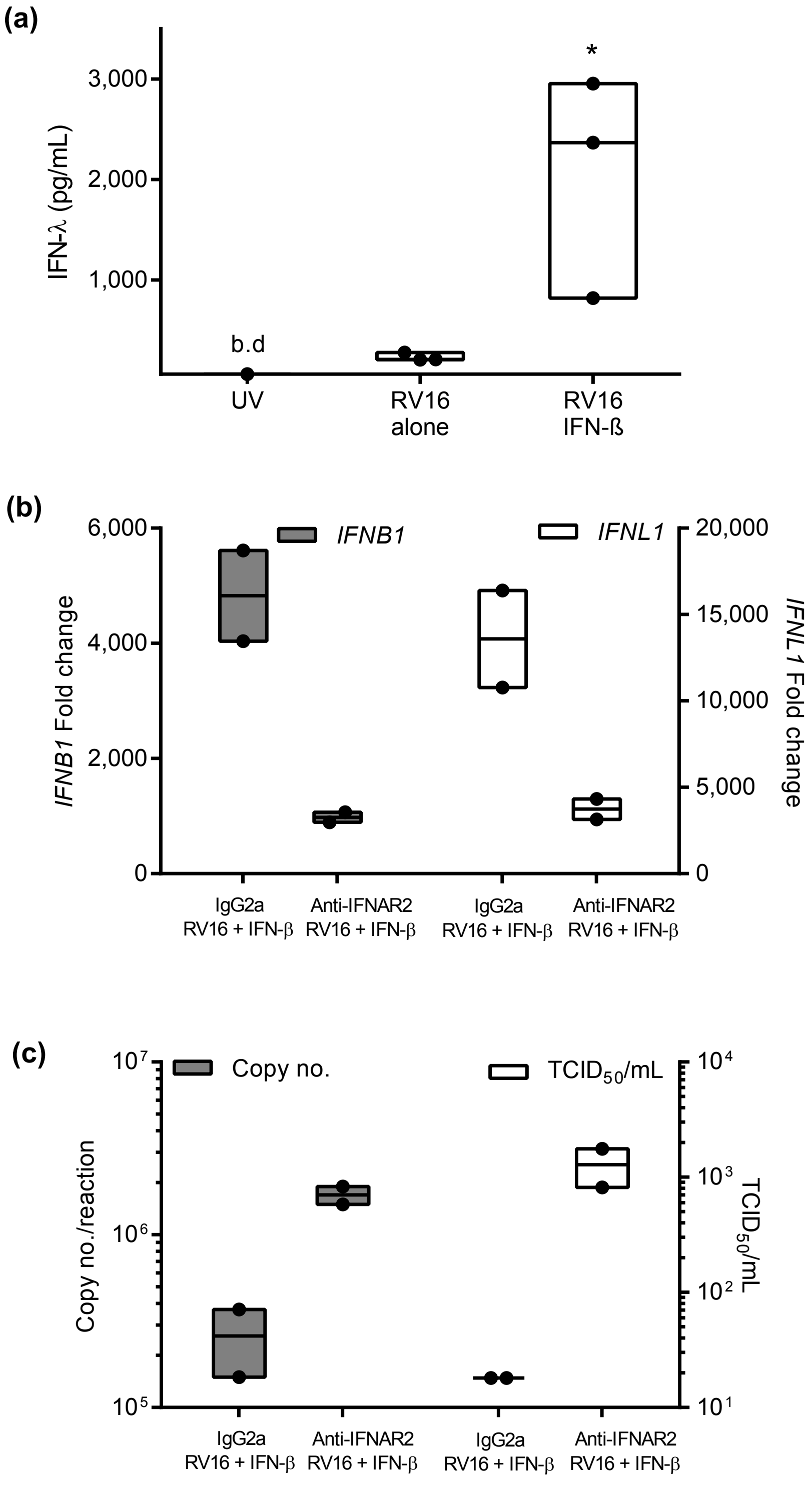

Fig. S4. IFN-β treatment and RV16 exposure of CBMCs and type I IFN receptor blockade. CBMCs were exposed to RV16 MOI 7.5 with IFN-β (100 IU/mL) and cell-free supernatants collected 24 hours post infection for protein quantification by ELISA. (**a**) IFN-λ protein expression, n=3, $*P \leq 0.05$ versus UV-RV16. CBMCs were pre-treated with anti-IFNAR2 antibody (1 μg/mL) or IgG2a isotype (1 μg/mL) prior to RV16 MOI 7.5 or UV-RV16 MOI7.5 infection in the presence of IFN-β. Twenty-four hours post infection cell pellets were harvested for gene expression and viral RNA by RT-qPCR and cell-free supernatants were harvested for infectious virus particles by $TCID_{50}$ assay. (**b**) *IFNB1* and *IFNL1* mRNA expression. (**c**) RV16 copy number and $TCID_{50}$/mL. n=2. Floating bars represent the median with min and max values, b.d. below limit of detection.

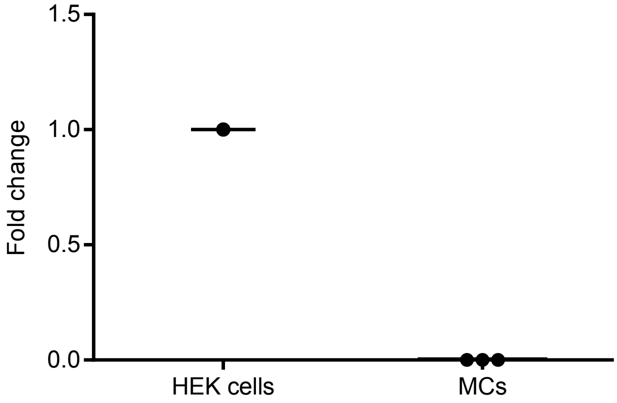

Fig. S5. CDHR3 expression following RV16 infection of LAD2 mast cells. MCs were exposed to RV16 at MOI 0.3, 3 or 7.5 or UV-RV16 MOI 7.5. Twenty-four hours post infection cell pellets were harvested for gene expression by RT-qPCR. Fold change (where an amplification product was detected) was expressed relative to HEK-293 cells stably transfected with *CDHR3*. LAD2 MCs n=5. MOI, multiplicity of infection.
